# Aspergillus flavus Infection of Lower Limb in an Immune-Competent Patient

**DOI:** 10.7759/cureus.9342

**Published:** 2020-07-22

**Authors:** Terrence Jose Jerome, Lakshmi Kandasamy, Thirumagal Kuppusamy Terrence, Suresh Bhalaji, Bhuvaneswari Shanmugasundaram

**Affiliations:** 1 Orthopaedics, Hand and Reconstructive Microsurgery, Olympia Hospital and Research Centre, Trichy, IND; 2 Microbiology, K. A. P. Viswanatham Medical College, Trichy, IND; 3 Reproductive Medicine, Olympia Hospital and Research Centre, Trichy, IND; 4 Urology, Dhanalakshmi Medical College, Perambalur, IND; 5 Pharmacology, Dhanalakshmi Medical College, Perambalur, IND

**Keywords:** aspergillus flavus, lower limb, infection, immunocompetent

## Abstract

Isolated aspergillus infection of the lower limb is uncommon and needs more focus in terms of understanding the pathogenicity, ecological and geographical distribution, identification of species, management and follow-up. Aspergillus flavus involving the lower limb in an immunocompetent individual is a rare entity. Surgical management and antifungal therapy are the mainstays of treatment. We report a 44-year-old farmer who presented with right lower limb swelling of short duration, operated, diagnosed with isolated species of Aspergillus flavus, treated successfully with oral voriconazole to produce excellent wound healing and functional outcome at four years follow-up.

## Introduction

Aspergillus flavus is more common in the air for unclear reasons and causes aggressive and invasive aspergillosis [1]. Chronic granulomatous sinusitis, keratitis, cutaneous aspergillosis, wound infections, and osteomyelitis following trauma and inoculation are the known spectrum of its manifestations in immunocompromised patients [2]. The proposed theory of such infections in individuals and especially people working in soil and farms is its capability of infecting the insects which then produce mycotoxin especially in maize and peanuts farms which are potentially harmful to animals or humans [3]. The word ‘aflatoxin’ came from ‘Aspergillus flavus toxin ‘which is responsible for invasive aspergillosis and wound infections. A. flavus group contains nine species and two varieties thus making accurate species identification within Aspergillus flavus complex and remains difficult due to overlapping morphological and biochemical characteristics [4]. Primary cutaneous aspergillosis is commonly caused by Aspergillus flavus, Aspergillus fumigates, Aspergillus niger, Aspergillus terreus and Aspergillus ustus. This is quite distinct in immunocompromised patients [5]. We present a healthy farmer with clinical features of skin and soft tissue infections of undivulged etiology diagnosed with isolated species of Aspergillus flavus and treated with relapse-free.

## Case presentation

**Figure 1:**
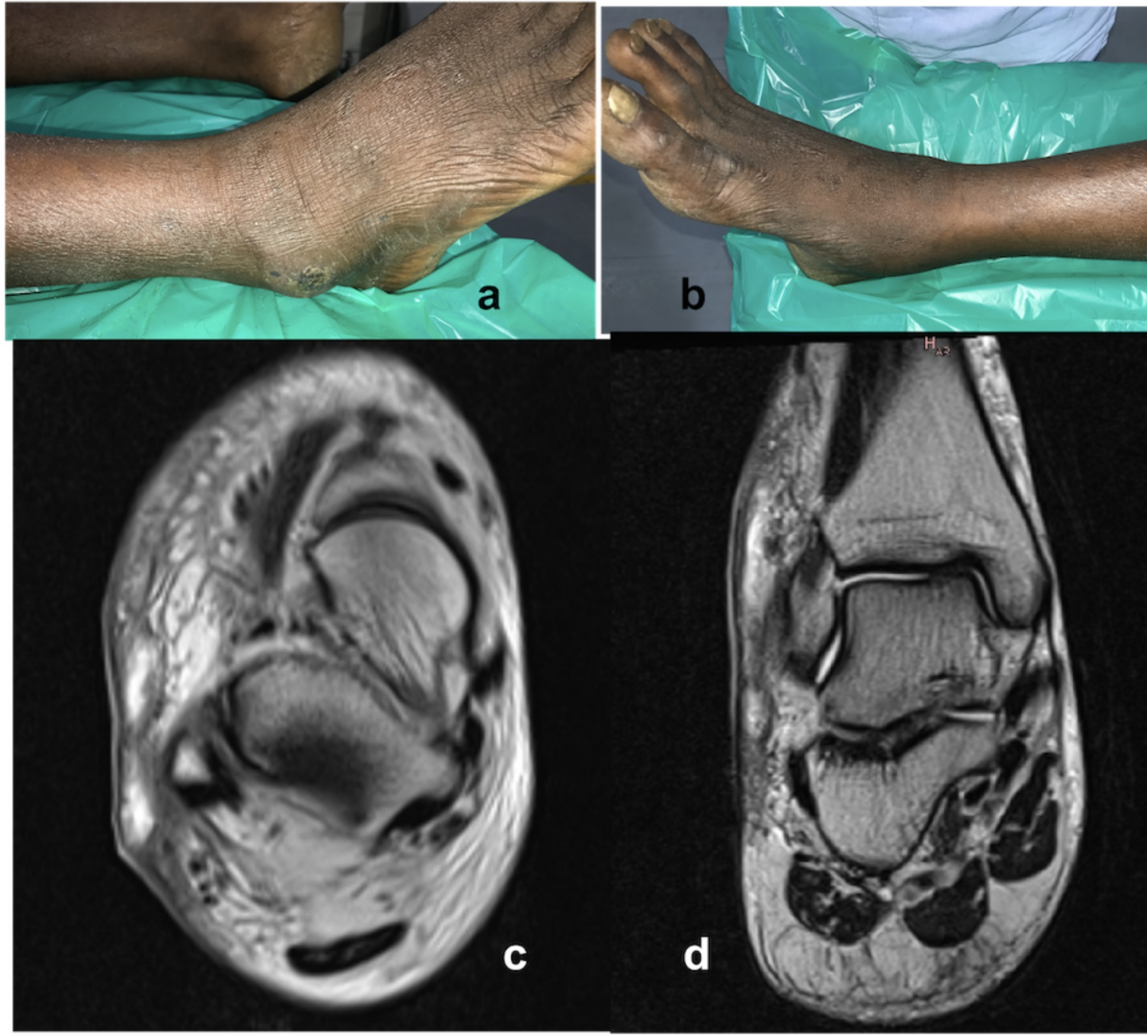
Clinical picture Clinical picture showing subcutaneous abscess over the lateral malleolus (a) and infection subsided picture of the medial side (b). MRI images showing multiple small to large pockets of abscess collection in the subcutaneous plane on the lateral aspect of ankle (c,d).

A 44-year-old farmer presented to the emergency department with pain, intermittent low-grade fever, swelling right lower limb of two weeks duration. Unable to recollect the mode of injury or trauma to this right ankle while working in his farm fields, he had diffuse swelling over both malleoli, foot, and lower leg which was also warm and tender. Clinical findings suggested acute cellulitis. C-reactive protein (CRP) was 22 mg/L (normal <10 mg/L) and erythrocyte sedimentation rate (ESR) was 50 mm/hr. Blood sugar level was 90 mg/dL. The rest of the blood investigations, urine tests, liver, and kidney function tests were found normal. Human immunodeficiency virus (HIV) test was negative. Radiographs of the ankle and leg were normal. Magnetic resonance imaging (MRI) of the right ankle showed multiple pockets of the subcutaneous collection over the lateral malleolus (Figure 1). Considering his clinical and radiological findings of skin and soft tissue infections (cellulitis), he was started on empirical intravenous antibiotics (piperacillin/tazobactam, amikacin, and clindamycin) and paracetamol. The pain and swelling did not subside with intravenous antibiotics. Further, he developed multiple small to large pockets of localized abscess over the lateral malleolus (Figure 1).

**Figure 2:**
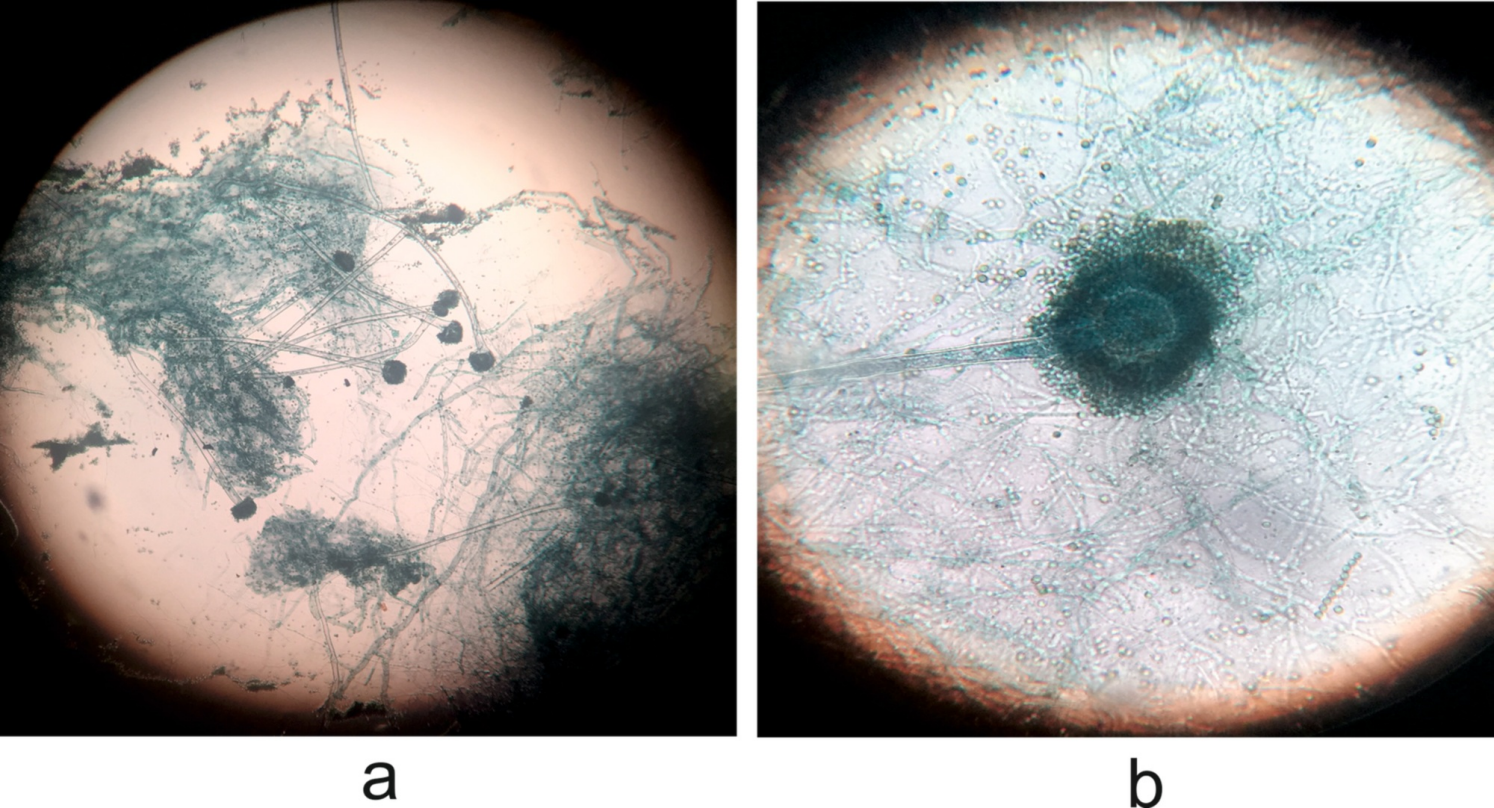
Microscopic findings Direct microscopic examination showing septate hyphal elements (a) and lactophenol cotton blue (LPCB) mounts revealed septate branching hyphal structures with conidiophores (b). (Original magnification x40)

Hence surgical debridement was planned. The pus and debrided tissues were sent for histopathological evaluation. Gram stain showed no bacteria and Ziehl-Neelsen stain, Kinyoun acid-fast stain was negative for acid-fast bacilli. Direct microscopic examination showed a lot of septate hyphal elements and fungal culture revealed white cotton wooly fluffy structures with yellowish-green powdery deposits. Lactophenol cotton blue (LPCB) mounts founded septate branching hyphal structures with conidiophores ending in an enlarged vesicle bearing conidiospores all around the vesicle (Figure 2).

Following this, the patient had a chest CT scan and was found normal. The counts of CD3+, CD4+, CD8+ lymphocytes were also found normal. He was started on oral voriconazole 200 mg/day twice a day for six weeks duration. He tolerated the medications and the wound healed without further complications. The patient was symptom-free at 48 months follow-up.

## Discussion

Aspergillus species have emerged as an important cause of life-threatening infections especially in immunocompromised patients [6]. Aspergillosis may occur as either primary or secondary cutaneous infection. In primary cutaneous aspergillosis, the lesion occurs as a result of direct inoculation of Aspergillus spores at the site of injury following intravenous catheter, trauma, occlusive dressings and tapes, burns or surgery. In secondary cutaneous aspergillosis, the lesions occur due to hematogenous dissemination from a primary focus such as the lungs or to contiguous spread to the skin from underlying infected structures or from a neighboring cavity such as the maxillary sinus. The classic clinical presentations are the presence of violaceous macules, papules, hemorrhagic bullae, ulcerations with central necrosis, pustules, or subcutaneous abscesses [7].

The clinical manifestations of our case resembled skin and soft tissue infections (SSTIs). The minimum diagnostic criteria for SSTI are erythema, edema, warmth, and pain or tenderness which was the clinical presentation of our patient in the emergency room. Approximately 70% to 75% of all cases involving the lower leg region are managed in the outpatient setting with oral antibiotics and anti-inflammatory medications. Empirical treatment (intravenous) is started for severe infections with complications to target staphylococcal and streptococcal species (especially S. aureus and S. pyogenes). Gram-negative or anaerobic colonization can be treated with cephalosporins, aminoglycosides, and clindamycin for enhanced coverage of group A streptococcal species. The clinical presentations, etiology, and progressing nature of presentation even after intravenous antibiotics narrowed us to two options. Fungal infection or insidious onset of serious life-threatening infections, such as necrotizing fasciitis. Differentiating between these two became clinically challenging. Poor response to intravenous antibiotics, absence of blisters, persistent fever, tachycardia, fatigue, hemorrhagic bullae, crepitus skin crackling, and slow progression of abscess in a clinically stable farmer ruled out necrotizing fasciitis [8]. Besides surgical drainage, debridement, and histopathological evaluation showed isolated species of Aspergillus flavus. Timely diagnosis and differentiation between life-threatening necrotizing fascitis and fungal aspergillus infection in an immunocompetent individual played a vital role in treating this patient without any complications.

Various species of Aspergillus are distributed in nature and only a few are pathogenic to man. Aspergillus fumigatus and Aspergillus flavus are the common offenders in systemic infections [5,9]. Skin injury or trivial trauma is found to be a predisposing factor for invasive aspergillosis. Our patient, who is a farmer, might have had trivial or unnoticed skin abrasions to his right ankle during his long hours of work in the fields. Aflatoxin (Aspergillus flavus toxin) could have been inoculated into the subcutaneous tissue causing cutaneous aspergillosis added to secondary bacterial infections. A hot and humid climate in India could have also increased the occurrence of such cutaneous lesions [7]. The most common factors predisposing to aspergillosis include granulocytopenia, hematological disorders, diabetes mellitus, neonatal period, local tissue injury, and primary or acquired diseases which cause immunosuppression. Immunocompetent patients can rarely develop cutaneous aspergillosis at the site of surgical wounds or by traumatic inoculation. If this happens then the sites vulnerable could be at the intravenous access catheter sites, traumatic or surgery wounds, and occlusive dressings in burns cases [9,10]. Clinical features may mimic tuberculosis where investigations such as CT scan chest and MRI could help in differentiating it. Periodic acid Schiff (PAS) stain, methenamine-silver-stain, galactomannan test and fungal culture are essential and specific for diagnosing Aspergillus species [11].

Voriconazole is recommended as an effective and primary therapy for the successful treatment of extrapulmonary and disseminated aspergillus infection [1]. It is widely distributed in mammalian tissues, with cerebrospinal fluid (CSF) levels of ∼50% in plasma levels. The elimination half-life of ∼6 h warrants twice-daily dosing. It is hepatically metabolized, with only 5% of the drug appearing unchanged in the urine. It is initiated with a loading dose of 6 mg/kg IV every 12 h for two doses, followed by 4 mg/kg every 12 h. These dosages are greater than those routinely administered for oral therapy (200 mg every 12 h). Oral therapy can be approximated to the standard IV dosage by using 4 mg/kg/dose rounded up to convenient pill sizes for six to twelve weeks depending upon the illness. Alternative anti-fungal agents L-Amphotericin B, posaconazole, itraconazole, or an echinocandin are used depending upon the prognosis, severity, and nature of the illness [1,2]. Our patient received 200 mg/day twice-daily dosing for six weeks and his wound healed without complications.

## Conclusions

A high index of suspicion and caution should be implemented in treating skin and superficial infections of lower limbs. Considering the ubiquity, overall pathogenicity, genomics, and taxonomy of aspergillosis, it should be borne in mind that if there is no perceptible clinical improvement of individuals with skin and soft tissue infections with or impending abscess, surgical drainage and debridement should be the next step in the treatment ladder along with cytological, fungal culture, and mycological evaluation to identify the species and varieties. Despite the normal hematological, biochemistry, and immunology tests, normal individuals are prone to develop cutaneous Aspergillus flavus infection because of its prevalence and virulence. Voriconazole is the drug of choice. Treatment should be judicious and meticulous.
